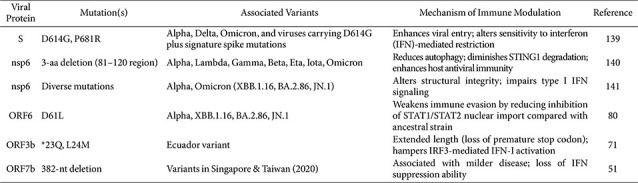# Modulation of Host Innate Immune Response by Highly Pathogenic Human Coronaviruses during Viral Infection

**DOI:** 10.4014/jmb.2602.02038

**Published:** 2026-05-25

**Authors:** Yu-Jin Kim, Su Jin Lee, Wooseong Lee, Seong-Jun Kim, Dae-Gyun Ahn

**Affiliations:** 1 Center for Infectious Disease Vaccine and Diagnosis Innovation, Therapeutics and Biotechnology division, Korea Research Institute of Chemical Technology, Daejeon 34114, Republic of Korea; 2 Department of Microbiology, School of Medicine, Kyungpook National University, Daegu 41944, Republic of Korea; 3 Untreatable Infectious Disease Institute, Kyungpook National University, Daegu 41944, Republic of Korea; 4 Institute of Endemic Disease, Seoul National University Medical Research Center, Seoul 03080, Republic of Korea

**Keywords:** Coronavirus, COVID-19, Innate immunity, Immune evasion, Immune modulation

## Abstract

Highly pathogenic human coronaviruses, including SARS-CoV, SARS-CoV-2, and MERS-CoV have emerged as significant public health threats due to their ability to cause widespread outbreaks and pandemics. These viruses induce dysregulated inflammatory responses, typified by cytokine storms that drive extensive tissue damage in pulmonary and extrapulmonary systems, leading to acute respiratory distress syndrome (ARDS) and multi-organ failure. These pathological outcomes are driven by sophisticated mechanisms that manipulate host immune pathways and evade innate and adaptive immune surveillance. The innate immune system plays a pivotal role in the early detection and control of viral infections through mechanisms such as cytoplasmic RNA sensors, Toll-like receptors, interferon signaling, and inflammasome activation. However, these coronaviruses effectively exploit and subvert these processes, suppressing antiviral defenses while amplifying inflammatory cascades. This review delineates the molecular and cellular strategies employed by these pathogens to evade immune recognition and exacerbate immune-mediated tissue injury. Understanding these processes is fundamental for guiding the development of targeted antiviral interventions, immunomodulatory therapeutics, and robust strategies to mitigate the impact of future coronavirus pandemics.

## Introduction

Coronaviruses are enveloped, positive-sense single-stranded RNA viruses belonging to the family *Coronaviridae* [1]. To date, seven human coronaviruses (HCoVs) have been identified: HCoV-229E, HCoV-OC43, HCoV-NL63, HCoV-HKU1, severe acute respiratory syndrome coronavirus (SARS-CoV), Middle East respiratory syndrome coronavirus (MERS-CoV), and SARS-CoV-2 [2]. The first four (HCoV-229E, HCoV-OC43, HCoV-NL63, and HCoV-HKU1) are referred to as common HCoVs. These HCoVs typically cause mild upper respiratory tract infections, such as the common cold, and are generally self-limiting in healthy individuals [2]. In contrast, the latter three (SARS-CoV, MERS-CoV, and SARS-CoV-2) are classified as highly pathogenic human coronaviruses due to their potential to cause severe and often fatal diseases in humans. These viruses primarily infect the lower respiratory tract, leading to severe clinical manifestations such as acute lung injury (ALI), acute respiratory distress syndrome (ARDS), sepsis, and multi-organ failure, often driven by dysregulated and excessive inflammatory responses [3]. SARS-CoV and MERS-CoV emerged in 2002 and 2012, respectively, sparking global health emergencies and significantly increasing scientific interest in the coronavirus family [4]. Most notably, SARS-CoV-2, which emerged in late 2019, is the first human coronavirus known to cause a pandemic, coronavirus disease 2019 (COVID-19) [3]. Distinguished by its high transmissibility and broad spectrum of clinical manifestations, COVID-19 has had a profound global impact, affecting not only the respiratory system [5] but also cardiovascular [6], neurological [7], and gastrointestinal functions [8] in many patients.

The innate immune response is the host’s first line of defense against invading pathogens. Unlike the adaptive immune response, the innate immune system recognizes common features of pathogens known as pathogen-associated molecular patterns (PAMPs), via pattern recognition receptors (PRRs) [9]. During viral infection, PRRs detect specific viral components, such as viral RNA, DNA, or replication intermediates. Recognition of PAMPs by PRRs initiates the host’s innate immune response, activating signaling cascades that culminate in the production of type I and III interferons (IFNs), along with proinflammatory cytokines and chemokines [10]. Most vertebrate PRRs are classified into several classes including retinoic acid-inducible gene I (RIG-I)-like receptors (RLRs), Toll-like receptors (TLRs), nucleotide-binding oligomerization domain (NOD)-like receptors (NLRs), C-type lectin receptors (CLRs), absent in melanoma-2 (AIM2)-like receptors (ALRs), and cyclic GMP-AMP synthase (cGAS) [9, 11]. Once the PRRs bind their respective ligands, adaptor molecules are recruited, thereby initiating downstream signaling cascades that accelerate proinflammatory responses [9].

While innate immune activation is essential for controlling viral infections, excessive or prolonged stimulation can lead to systemic inflammation and collateral tissue damage. Therefore, the innate immune response must be tightly regulated to ensure effective viral clearance without harming host tissues. However, many viruses, including coronaviruses, have evolved immune evasion strategies to evade innate immunity, suppressing antiviral signaling and allowing them to subvert host defenses and establish infection [12]. Coronavirus proteins frequently display multifaceted activities, modulating distinct components of the immune response in parallel. These overlapping and coordinated actions enhance the overall efficiency of immune suppression. Despite significant progress in coronavirus research, the complex mechanisms by which highly pathogenic human coronaviruses evade innate immune detection and manipulate host responses remain incompletely understood.

In this review, we summarize current knowledge of the immune evasion strategies employed by highly pathogenic human coronaviruses, with particular emphasis on the interconnected nature of these mechanisms and the multiple, overlapping nodes targeted simultaneously by viral proteins.

Unlike previous reviews that primarily describe individual viral proteins or specific immune pathways, this review aims to provide an integrative framework that connects diverse innate immune evasion mechanisms into a unified network. We particularly focus on identifying central host vulnerability nodes that are recurrently targeted by multiple viral proteins and on delineating the hierarchical and temporal coordination of immune evasion strategies, including early suppression of interferon responses and later activation of inflammatory pathways. In addition, we provide a comparative analysis across SARS-CoV, MERS-CoV, and SARS-CoV-2 to highlight both conserved and virus-specific mechanisms, thereby offering a systems-level perspective on coronavirus immune modulation. It should be noted that, compared to SARS-CoV and especially SARS-CoV-2, the molecular mechanisms underlying MERS-CoV immune evasion remain less extensively characterized. This imbalance in available data represents an important limitation in the field and underscores the need for further mechanistic studies on MERS-CoV-host interactions. Despite these limitations, we aimed to provide as balanced a comparison as possible by integrating available findings from all three coronaviruses and highlighting both shared and distinct immune evasion mechanisms.

### Coronavirus Life Cycle and Immune Evasion Strategies

**Fig. 1:**
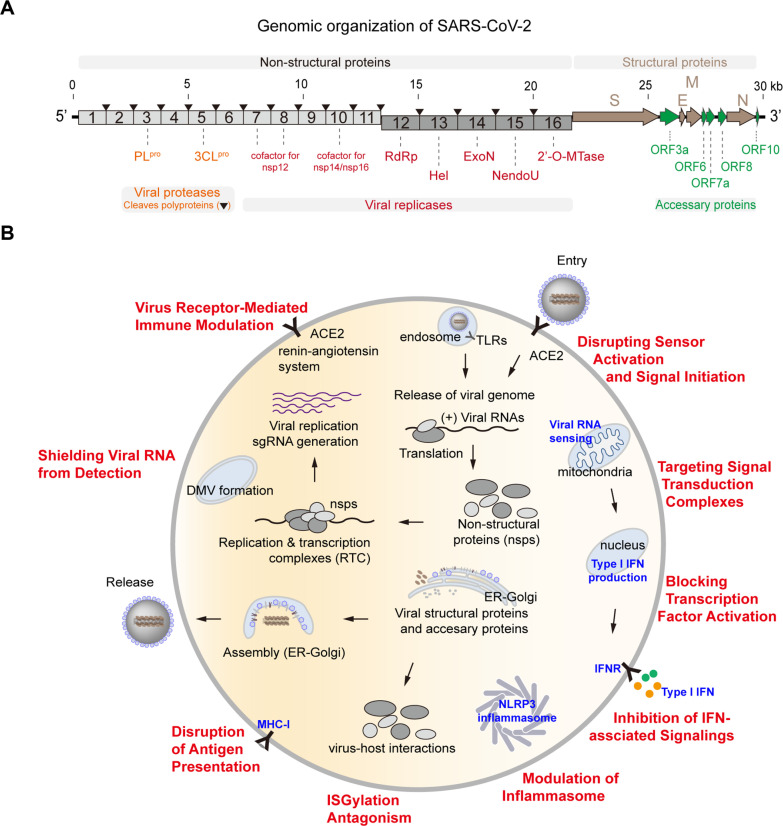
Coronavirus life cycle and strategic immune evasion pathways. (A) Genomic organization of SARS-CoV-2. Coronaviruses possess an unusually large RNA genome, approximately 30 kilobases in length. The 5′ end of the genome encodes non-structural proteins within open reading frames ORF1a (light brown) and ORF1b (dark brown), while the 3′ end encodes structural proteins (navy), S, E, M, and N, along with various accessory proteins (green). Non-structural proteins are produced through proteolytic cleavage of polyproteins 1a and 1ab by two viral proteases: nsp3 and nsp5. The functional roles of several viral proteins are illustrated accordingly. (B) Strategic immune evasion pathways of coronaviruses during infection. Viral entry is mediated through receptor-dependent membrane fusion and endocytosis. Replicase proteins initiate the formation of RTCs, which drive genome replication and the synthesis of sgRNA encoding structural and accessory proteins. Structural proteins are trafficked to the ER and Golgi apparatus membranes, where virion assembly occurs through encapsidation of genomic RNA. Progeny virions are released via exocytosis. Throughout the replication cycle, host antiviral responses are activated (blue), while viral proteins employ diverse strategies (red) to counteract these defenses through targeted virus-host interactions that suppress immune signaling and facilitate immune evasion.

Coronaviruses, including SARS-CoV, SARS-CoV-2, and MERS-CoV, enter host cells through receptor-mediated membrane fusion or endocytosis. Following cellular entry, coronaviruses initiate translation of polyproteins 1a and 1ab, which encode approximately 16 non-structural proteins (nsps) (Fig. 1A). These polyproteins are cleaved by two viral proteases, nsp3 (PLpro) and nsp5 (3CLpro), to release replicase components that assemble into replication/transcription complexes (RTCs). Key replicase proteins include nsp12 (RNA-dependent RNA polymerase, RdRp), nsp13 (helicase), nsp14 (exoribonuclease, ExoN), nsp15 (endoribonuclease, NendoU), and nsp16 (2’-O-methyltransferase), supported by cofactors such as nsp10 (for nsp14 and nsp16) and nsp7/nsp8 (for nsp12). During viral replication, the resulting subgenomic RNAs (sgRNA) function as mRNAs encoding structural and accessory proteins (*e.g.*, ORF3a, ORF6). A comprehensive overview of the SARS-CoV-2 life cycle has been provided in a recent review by Steiner *et al*. [13].

Throughout the coronavirus life cycle (Fig. 1B), the viral genome transitions through three distinct forms, single-stranded genomic RNA (ssRNA), sgRNAs, and double-stranded RNA (dsRNA), which serve as intermediates during replication and transcription. Viral RNAs are recognized by PRRs, including RLRs in the cytosol and TLRs in endosomes. RIG-I primarily detects short dsRNA, whereas melanoma differentiation-associated protein 5 (MDA5) preferentially senses long dsRNA [14]. During SARS-CoV-2 infection, activation of RIG-I and MDA5 in lung epithelial cells triggers robust innate immune responses [15-18]. Notably, higher expression levels of RIG-I and MDA5 in children may contribute more strongly to efficient early antiviral sensing compared to adults [19]. RIG-I has also been implicated in MERS-CoV-induced inflammatory responses [20].

Among TLRs, TLR3 and TLR7 have been implicated in the recognition of viral RNA during SARS-CoV and SARS-CoV-2 infections [21, 22]. Recent studies suggest that TLR2 may detect the SARS-CoV-2 envelope (E) protein, initiating inflammatory signaling [23]. Additionally, the Spike (S) protein of SARS-CoV-2 activates inflammatory pathways via TLR2 and TLR4 [24-26], while TLR7 is essential for sensing the nucleocapsid (N) protein of MERS-CoV [27].

Upon activation by viral RNA, RIG-I and MDA5 undergo conformational changes that promote multimerization of their caspase-recruitment domains (CARDs), facilitating CARD–CARD interactions with mitochondrial antiviral-signaling protein (MAVS), the key adaptor in RLR signaling [28]. MAVS is anchored to mitochondria, mitochondrial-associated membranes (MAMs), and peroxisomes via its transmembrane domain. It propagates signals to TANK-binding kinase 1 (TBK1) and IκB kinase-ε (IKKε), which subsequently activate transcription factors IRF3 and IRF7. Together with nuclear factor-κB (NF-κB), these factors drive the expression of type I and III interferons and a range of inflammatory genes [28]. Depending on the adaptor recruited, TLR signaling is categorized into MyD88-dependent pathways, which mainly induce inflammatory cytokines, and TRIF-dependent pathways, which drive both type I interferon production and inflammatory cytokine responses [29].

However, many of coronavirus proteins actively antagonize host immune defenses through diverse mechanisms, including disruption of PRR activation, interference with signaling complexes, inhibition of transcription factor activity, suppression of interferon signaling, modulation of inflammasome function, impairment of antigen presentation, and evasion of antiviral pathways such as ISGylation (Fig. 1B). Importantly, these immune evasion strategies are tightly integrated with specific stages of the viral life cycle and are deployed in a coordinated manner to subvert host immunity. Elucidating these interactions is critical for identifying therapeutic targets and developing effective antiviral strategies.

### Functional Strategies of Coronavirus Immune Modulation

**Shielding Viral RNA from Detection:**

During replication of the viral genome, coronaviruses generate dsRNA intermediates that can be detected by host RNA sensors. To evade activation of the innate immune system, coronaviruses employ multiple strategies to conceal these replication intermediates (Fig. 1B). Several coronavirus nonstructural proteins, including nsp3, nsp4, and nsp6, form double-membrane vesicles (DMVs) by hijacking the host endoplasmic reticulum (ER) membrane, thereby shielding viral replication complexes from cytoplasmic RNA sensors [30-32]. In addition, coronaviruses modify their sgRNA, which function as mRNAs, by generating a cap structure at the 5’ end, allowing efficient translation by the host machinery while avoiding recognition by RNA sensors. The sgRNA capping process involves several viral nonstructural proteins: nsp13 exhibits 5′ RNA triphosphatase activity, nsp14 functions as an N7-methyltransferase, and nsp16 acts as a 2′-O-methyltransferase [33, 34]. Furthermore, degradation of viral dsRNA has been identified as another immune evasion mechanism. Using a recombinant SARS-CoV-2 strain harboring an nsp15 mutation, it has been demonstrated that the SARS-CoV-2 nsp15 endoribonuclease degrades viral dsRNA, thereby reducing dsRNA accumulation and limiting activation of antiviral immune responses [35]. In addition to degradation, viral proteins can directly mask viral RNAs. For example, the MERS-CoV ORF4a protein functions as a dsRNA-binding protein that masks viral dsRNA, thereby preventing its recognition by TLR3 and RIG-I and suppressing subsequent antiviral responses [36, 37].

**Disrupting Sensor Activation and Signal Initiation:**

**Fig. 2:**
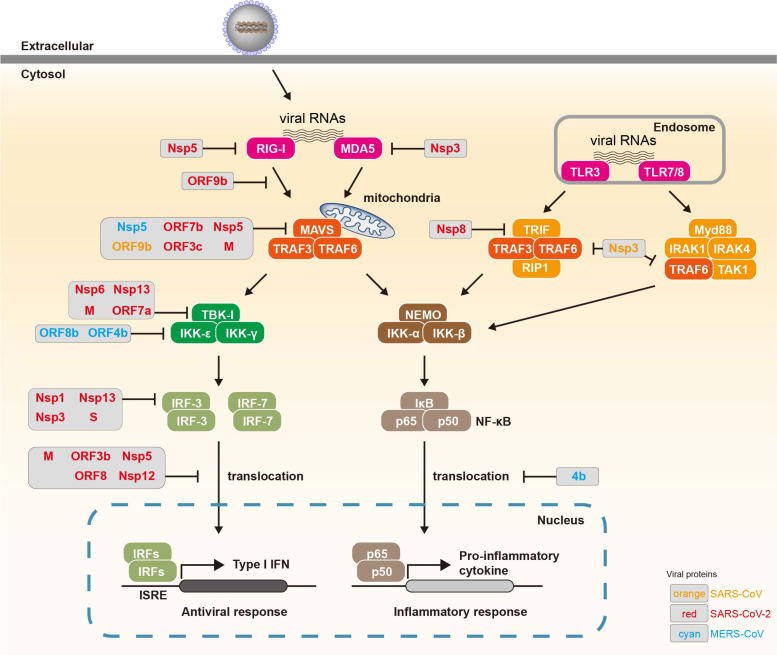
Viral suppression of innate immunity via RNA-sensing pathways. Innate immune responses are initiated upon sensing viral RNAs. These RNAs are detected either by cytosolic RNA sensors, known as RLRs, or TLRs, specifically TLR3, TLR7, and TLR8. Upon recognition, RLRs activate signaling cascades via the MAVS, leading to the activation of IRF3 or IRF7 and subsequent induction of type I IFNs (*e.g.*, IFN-β). In parallel, TLR-mediated signaling involves adaptor proteins such as NF-κB essential Modulator (NEMO) and transcription factors like NF-κB, resulting in the production of pro-inflammatory cytokines (*e.g.*, IL-6, TNF-α). To evade or suppress host immune defenses, viral proteins strategically interfere with each step of these signaling pathways. The specific viral proteins involved in these inhibitory mechanisms are indicated at their respective points of action.

RLRs are primary targets of initial suppression of innate immune responses (Fig. 2). Biochemical analysis revealed that SARS-CoV-2 nsp5 cleaves the 10 most N-terminal amino acids from RIG-I, thereby preventing it from activating MAVS [38]. Recent reports have described that SARS-CoV and SARS-CoV-2 N proteins interfere with the interaction between RIG-I and its downstream factor, tripartite motif protein 25 (TRIM25), suppressing RIG-I-mediated IFN production [39-41].

MAVS is a critical target for immune evasion due to its central role in signal transduction mediated by RIG-I and MDA5. SARS-CoV-2 nsp5 promotes the ubiquitination and proteasome-mediated degradation of MAVS, thereby attenuating innate immune responses [38]. Interestingly, Huizen *et al*. demonstrated that MAVS cleavage by nsp5 occurs exclusively in MERS-CoV-infected cells, but not in SARS-CoV-2-infected cells [42]. SARS-CoV ORF9b facilitates MAVS degradation to suppress innate immune responses [43], whereas SARS-CoV-2 ORF9b disrupts the interaction between RIG-I and MAVS [44], thereby inhibiting type I IFN production. Furthermore, SARS-CoV-2 ORF9b localizes to mitochondria and associates with the translocase of the outer membrane 70 (TOM70), leading to suppression of the type I IFN signaling pathway [45, 46]. ORF3c also can interact with MAVS and induce its C-terminal cleavage by caspase-3, thereby blocking the interaction between RIG-I and MAVS [47, 48]. Additionally, the SARS-CoV-2 M protein directly targets MAVS, thereby impairing its aggregation and recruitment of downstream signaling components [49]. The M protein can also interact with cytosolic viral RNA sensors, RIG-I, MDA5, MAVS, and TBK1, disrupting the formation of the multiprotein complex required for effective signaling, ultimately inhibiting type I and III interferon production [50]. SARS-CoV-2 ORF7b has also been identified, through IFNβ promoter screening, as a major suppressor of MAVS-induced interferon production, but its direct molecular targets remain elusive [51].

While research on RLR-mediated sensing of coronaviruses is extensive, emerging evidence highlights the importance of TLRs in viral recognition and immune modulation. SARS-CoV-2 has been reported to modulate TLR signaling pathways, particularly TLR3, TLR7, and TLR8, to attenuate type I interferon responses [52-54] . Viral proteins such as nsp13 and ORF9b have been implicated in the suppression of TLR-mediated NF-κB and IRF activation [55, 56]. Furthermore, studies using TLR7 agonists have demonstrated that the SARS-CoV nsp3 (PLpro) removes Lys63-linked polyubiquitin chains from TRAF3 and TRAF6, leading to inhibition of TLR7-mediated signaling [57]. Although the current body of evidence is less comprehensive than that for RLRs, *in vivo* studies and patient-derived data suggest that TLR dysfunction may contribute to delayed interferon production and hyperinflammatory responses observed in severe COVID-19 [58, 59]. Future studies focusing on TLR-specific antagonism or activation may reveal new therapeutic avenues, especially in the context of SARS-CoV-2 variants exhibiting differential TLR modulation.

**Targeting Signal Transduction Complexes:**

As a downstream signaling factor of MAVS, TBK1 has been reported as a key target for viral immune evasion. SARS-CoV-2 M protein promotes the degradation of TBK1 via K48-linked ubiquitination [60]. Overexpression of the M protein reduces TBK1 levels, thereby impairing downstream signaling cascades, including the formation of the TRAF3–TANK–TBK1–IKKε complex and the activation of IRF3, ultimately suppressing type I IFN expression [60]. SARS-CoV-2 nsp6 directly binds to TBK1 and inhibits the phosphorylation of IRF3 [61], a crucial transcription factor for type I IFN production, while nsp13 not only interacts with TBK1 to block its phosphorylation and suppress activation but has also been reported to inhibit the phosphorylation of both TBK1 and IRF3, ultimately leading to downregulation of type I IFN expression [56, 61]. MERS-CoV ORF4b directly interacts with TBK1 and IKKε, disrupting the association between MAVS and IKKε, which leads to reduced phosphorylation of IRF3 and diminished IFN-β production [62]. Notably, overexpression of MERS-CoV ORF4b also suppresses IFN-β expression triggered by IRF3 and IRF7 by an unknown mechanism [62]. MERS-CoV ORF8b interferes with the IKKε–HSP70 interaction, thereby preventing IKKε activation and subsequent IFN-β production [63]. Furthermore, overexpression of SARS-CoV-2 ORF7a reduces TBK1-mediated IFN-β promoter activity and decreases TBK1 protein levels, thereby impairing IRF3 phosphorylation, whereas viral proteins ORF7b, ORF8, ORF9b, ORF14, and 3CLpro fail to exert such effects, and the precise underlying mechanism of ORF7a remains to be elucidated [44].

**Blocking Transcription Factor Activation:**

In the RIG-I signaling cascade, IRF3 functions as a critical downstream effector of TBK1, becoming activated through phosphorylation. As such, IRF3 serves as a potential target for viral immune evasion. SARS-CoV-2 viral proteins block IRF3-mediated type I IFN production through diverse mechanisms, including proteasomal degradation of IRF3 by the S protein [64], direct interaction with the IRF association domain (IAD) of IRF3 by nsp13 [65], inhibition of IRF3 phosphorylation by nsp1 [66], and direct cleavage of IRF3 by nsp3 [67].

The nuclear translocation of IRF3 is critical for its function as a transcription factor. Interestingly, the nsp5 inhibits the nuclear translocation of phosphorylated IRF3 independently of its protease activity and promotes IRF3 degradation in cells overexpressing nsp5 [68, 69]. Its nuclear translocation is also impaired by nsp12 [70], without affecting its phosphorylation; by ORF3b with unknown mechanism [71]; by ORF8 through its interaction with HSP90B1 [72, 73]; and by ORF6 and M proteins through binding to the nuclear import factor karyopherin alpha 2 (KPNA2) [61, 74].

Alongside IRF3, IRF7 is also activated by TBK1 and IKKε, and together with NF-κB, they translocate into the nucleus to promote the expression of type I IFNs and other immune-related genes [28]. MERS-CoV ORF4b binds to karyopherin-α4, disrupting its interaction with the NF-κB p65 subunit, thereby blocking NF-κB nuclear translocation and suppressing type I IFN expression [75]. This broad suppression ultimately leads to the inhibition of type I and III IFN production.

**Evasion of IFN-Mediated Immune Responses:**

**Fig. 3:**
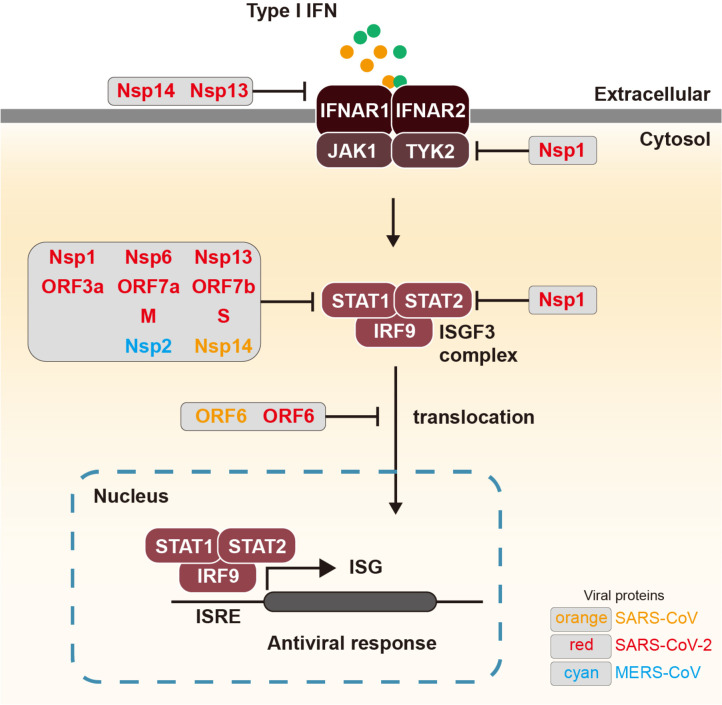
Viral interference with IFN-mediated signaling pathways. Type I IFNs, induced via RLRs, bind to their cognate receptors IFNAR1 and IFNAR2 on the cell surface. This engagement activates the JAK-STAT signaling pathway, leading to the phosphorylation and dimerization of STAT1 and STAT2. These phosphorylated STATs associate with IRF9 to form the ISGF3 complex, which translocates into the nucleus and binds to interferon-stimulated response elements (ISREs) in the promoters of target genes. This drives the transcription of ISGs, including OAS, PKR, ISG15, and MX1, thereby establishing an antiviral state. However, coronaviruses have evolved sophisticated strategies to evade this immune response by targeting and disrupting key steps in the signaling cascade.

**Receptor downregulation.** The end products of both RLR and TLR signaling pathways are type I and III IFNs, which trigger IFN-mediated immune responses through autocrine or paracrine signaling. Therefore, antagonizing the IFN signaling cascade represents a key immune evasion strategy employed by coronaviruses (Fig. 3). Notably, SARS-CoV-2 nsp13 and nsp14 have been shown to reduce the endogenous levels of the type I IFN receptor (IFNAR1), as demonstrated using full IFN-α4 and IFN-β promoter assay systems with overexpression of 30 SARS-CoV-2 proteins [76]. Specifically, nsp14 promotes the degradation of IFNAR1 via post-translational modifications [76].

**Blocking JAK-STAT signaling.** Upon ligand binding, the type I IFNAR activates the Janus kinases JAK1 and TYK2 through phosphorylation, which in turn phosphorylate the signal transducers and activators of transcription, STAT1 and STAT2, on specific tyrosine residues to initiate downstream signaling [77]. IFN-β promoter assays performed with overexpression of the complete set of SARS-CoV-2 proteins revealed that SARS-CoV-2 nsp1 depletes TYK2 by inhibiting host protein translation and depletes STAT2 through translational shutoff, thereby suppressing the expression of interferon-stimulated genes (ISGs) [66]. Consistent with these findings, other studies employing similar IFN-β promoter assays reported that multiple SARS-CoV-2 proteins including nsp1, nsp6, nsp13, ORF3a, ORF7a, ORF7b, M, and S proteins also interfere with STAT phosphorylation by suppressing the activity of upstream kinases such as TANK-binding kinase 1 (TBK1) and JAK1, collectively inhibiting phosphorylation of STAT1 and/or STAT2 [61, 74]. Mutational analyses further confirmed that the helicase activity of nsp13 is essential for its ability to suppress IFN signaling [78]. Moreover, ORF6 blocks IFN signaling by preventing the nuclear import of STAT1 and STAT2 through interaction with nuclear pore complex components Nup98 and Rae1 [79, 80]. Similarly, in SARS-CoV, ORF6 inhibits STAT1 nuclear translocation by binding to karyopherin α2 and β1 at the ER/Golgi membrane, thereby suppressing ISG expression [81, 82]. Additionally, SARS-CoV nsp14 and MERS-CoV nsp2 have been reported to impair IFN-α–induced phosphorylation of STAT1 and STAT3 in human epithelial cells [83].

**Evasion and Activation via Inflammasomes:**

**Fig. 4:**
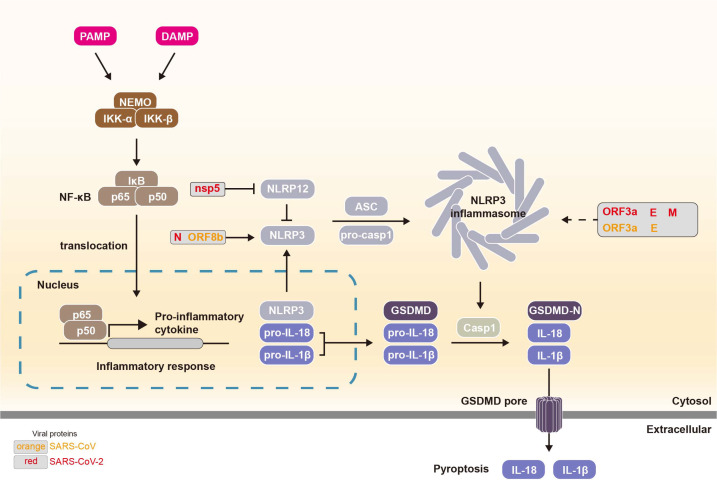
Viral protein-induced enhancement of inflammatory responses. Recognition of PAMPs and DAMPs initiates pro-inflammatory signaling pathways, leading to transcriptional upregulation of NLRP3, pro-IL-1β, and pro-IL-18. Upon sensing cellular stress or infection, NLRP3 oligomerizes and recruits the adaptor protein ASC and pro-casp1 to form the NLRP3 inflammasome complex. Inflammasome activation triggers the proteolytic activation of caspases including caspase-1 and, in some contexts, caspase-4/5 or caspase-11 which cleave GSDMD, pro-IL-1β, and pro-IL-18 into their mature, bioactive forms. The N-terminal fragment of GSDMD (GSDMD-N) translocates to the plasma membrane and forms pores, facilitating the extracellular release of IL-1β and IL-18 and inducing pyroptotic cell death. During viral infection, certain viral proteins can aberrantly activate inflammasomes, exacerbating inflammatory responses and contributing to immunopathology. In addition, viral proteins such as nsp5 can cleave NLRP12, a negative regulator of NLRP3, thereby indirectly promoting inflammasome activation.

Inflammasomes are large multi-protein complexes that play an essential role in the innate immune response to infections, particularly viral infections [84]. These complexes recognize PAMPs and damage-associated molecular patterns (DAMPs) [85], both of which trigger immune responses (Fig. 4). Among various types, the NLRP3 inflammasome is one of the most extensively studied and is activated in response to viral components [86]. Upon activation, NLRP3 recruits an apoptosis-associated speck-like protein containing a CARD (ASC) and caspase-1 to form a multiprotein complex [87]. Caspase-1 then cleaves pro-IL-1β and pro-IL-18 into their active forms, driving the secretion of these pro-inflammatory cytokines and initiating robust immune responses [88].

**Viral modulation of NLRP3 inflammasome.** The SARS-CoV-2 N protein interacts specifically with NLRP3 through a domain spanning amino acids 260–340, identified via truncated mutant analyses, but not with NLRP1, NLRC4, or AIM2, thereby functioning as a scaffold to manipulate host signaling [89]. Similarly, SARS-CoV ORF8b can bind the LRR domain of NLRP3 in macrophages, thereby activating the inflammasome and promoting strong inflammatory responses [90]. These interactions enhance NLRP3-ASC binding, facilitating inflammasome assembly and amplifying downstream inflammation [91-95]. In addition, both SARS-CoV and SARS-CoV-2 ORF3a contribute to NLRP3 activation [96, 97], directly promoting inflammasome assembly through K^+^ efflux and ASC recruitment [98]. Beyond NLRP3, NLRP12 acts as a negative regulator of pro-inflammatory cytokine release [99]. However, SARS-CoV-2 nsp5 cleaves NLRP12, thereby disrupting this regulatory function and enhancing inflammatory pathways [67].

In addition to direct inflammasome modulation, several viral proteins exert context-dependent effects. The SARS-CoV E protein has been shown to indirectly activate NLRP3 through its viroporin activity, which disrupts intracellular Ca^2+^ homeostasis [100]. More recently, the SARS-CoV-2 E protein was reported to suppress NLRP3 activation by reducing ER stress responses during early infection, but to promote activation under heightened inflammatory conditions, highlighting a stage- and context-dependent regulatory role [101]. Rather than acting as a simple immune suppressor, the E protein appears to exhibit a biphasic function that varies depending on the stage of infection and host immune status. In the early stage, it may restrain excessive inflammation to maintain a favorable environment for viral replication, whereas at later stages, as host immune activity increases, the virus may exploit the inflammasome pathway to induce tissue damage and immune imbalance.

In addition, the SARS-CoV-2 M protein has been implicated in NLRP3 activation in human monocyte-derived macrophages through its viroporin activity [98]. By contrast, the M protein of MERS-CoV is primarily associated with suppression of IFN signaling [102], indicating that even structurally conserved viral proteins have evolved distinct immunomodulatory strategies across coronaviruses. Thus, whereas the SARS-CoV-2 M protein may influence inflammation through inflammasome-related mechanisms, its MERS-CoV counterpart appears more closely tied to innate immune evasion.

**Two-sided perspectives of immune modulation.** As described above, coronavirus infection elicits both suppression and activation of inflammatory responses. Viral proteins can dampen innate immunity while simultaneously promoting inflammation, for example, SARS-CoV-2 ORF3a and E proteins activate the NLRP3 inflammasome to induce IL-1β [97, 100], whereas nsp1 blocks host protein synthesis and nsp13, nsp14, nsp15, and ORF6 antagonize IFN signaling [103, 104]. These opposing mechanisms allow coronaviruses to fine-tune host defenses for replication and immune evasion.

Disease outcome depends on the balance of these responses: moderate inflammation supports viral clearance and repair, but excessive activation drives cytokine storms and lung injury, while excessive IFN suppression permits uncontrolled replication and chronic damage [105, 106]. Both the magnitude and the timing of interferon responses are critical, early induction is protective, whereas delayed or systemic overactivation exacerbates pathology [105]. Thus, inflammation represents a double-edged sword, and understanding its dual regulation is essential for therapies that maintain an optimal balance between immune activation and suppression during coronavirus infection.

**Virus Receptor-Mediated Immune Modulation:**

There are various viral factors involved in modulating virus-induced innate immunity, some of which have not been categorized above. One such mechanism is virus-receptor-mediated immune modulation. The receptors involved in CoV infections include hACE2, hDPP4, DC-SIGN, and L-SIGN, each playing distinct roles in viral pathogenesis and immune modulation [107-109]. hACE2 (human angiotensin-converting enzyme 2) is the primary receptor for SARS-CoV and SARS-CoV-2 [110]. The S protein of these viruses binds to hACE2, enabling viral entry into host cells, particularly in the respiratory tract, kidneys, heart, and gastrointestinal system [111]. In the host, hACE2 plays a central role in regulating the renin-angiotensin system (RAS), which governs fluid balance, blood pressure, and inflammatory responses [112]. Under normal conditions, hACE2 converts angiotensin II into angiotensin, which counteracts the pro-inflammatory and vasoconstrictive effects of angiotensin II, promoting vasodilation, lowering blood pressure, and alleviating inflammation [113].

During SARS-CoV infection, viral binding to hACE2 leads to receptor internalization and downregulation, disrupting the protective RAS axis and exacerbating acute lung injury [114]. Similarly, in SARS-CoV-2–infected cells, interaction between hACE2 and the S protein promotes receptor degradation in lysosomes via the endocytic pathway [115, 116]. In both cases, hACE2 downregulation shifts the RAS toward Ang II–AT1R dominance, eliminating hACE2’s counter-regulatory effects and leaving angiotensin II unopposed [109]. This drives sustained NF-κB activation, creating a mechanistic link between viral entry and innate immune hyperactivation [117, 118]. As a result, surface receptor engagement propagates systemic inflammation and tissue injury in COVID-19 [119], ultimately weakening host defenses, delaying resolution, and heightening susceptibility to secondary infections and organ damage.

**ISGylation Antagonism:**

**Fig. 5:**
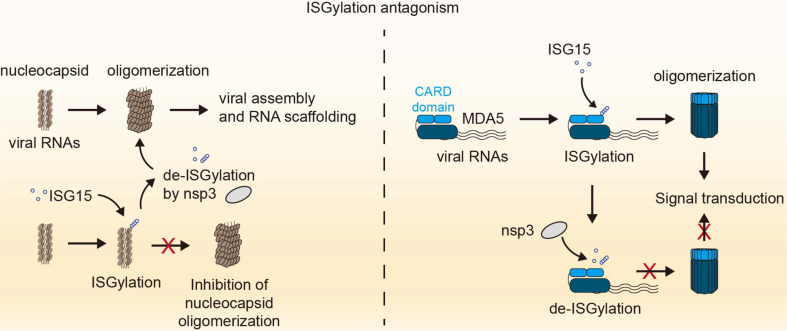
Viral antagonism of ISGylation-mediated host defense. During viral infection, ISGylation disrupts N protein oligomerization, impairing viral assembly; however, this antiviral mechanism is counteracted by SARS-CoV-2 nsp3, which removes ISG15 conjugates (left panel). MDA5 activation requires ISGylation at its CARD domain, promoting oligomerization and downstream type I IFN signaling. SARS-CoV-2 nsp3 de-ISGylates MDA5, thereby suppressing ISGylation-mediated immune responses (right panel).

Interferon-stimulated gene 15 (ISG15) is a well-characterized ubiquitin-like protein induced by type I interferons. It exerts antiviral effects through ISGylation, a process involving the covalent conjugation of ISG15 to viral proteins [120-123]. The oligomerization of the N protein in coronaviruses, including SARS-CoV-2, plays a critical role in viral assembly and RNA scaffolding (Fig. 5) [124]. During SARS-CoV-2 infection, HERC5 E3 ligase-mediated ISGylation targets the N protein, disrupting its oligomerization and thereby inhibiting viral replication [125]. Site-directed mutagenesis has revealed that the N proteins of SARS-CoV and MERS-CoV are also targeted for ISGylation through conserved lysine residues (K372 in MERS-CoV and K375 in SARS-CoV, corresponding to K374 in SARS-CoV-2) [125].

However, nsp3 of SARS-CoV, SARS-CoV-2, and MERS-CoV counteract this mechanism by acting as deconjugating enzymes for both ISGylation and ubiquitination [125], thereby enabling the virus to evade ISGylation-mediated antiviral activity [126]. Notably, MERS-CoV nsp3 exhibits deconjugating functions comparable to those of SARS-CoV-2, despite structural differences between the two enzymes, highlighting their shared role in modulating antiviral immune responses [127].

The CARD domains of RIG-I and MDA5 can undergo polyubiquitination, and ubiquitin-induced oligomerization is essential for the activation of type I IFN signaling cascades [128]. A recent study reported that MDA5, but not RIG-I, is activated via oligomerization followed by ISGylation at lysine residues K23 and K43 within its CARD domain (Fig. 5). In ISG15^−/−^ MEFs, ISG15 KO HeLa cells, and ISG15 KO HAP-1 cells, MDA5 activation was markedly attenuated. Interestingly, SARS-CoV-2 nsp3 specifically targets MDA5, but not RIG-I, leading to de-ISGylation and ultimately suppressing MDA5 activation [129].

**Disruption of Antigen Presentation:**

Additionally, SARS-CoV-2 undermines the host immune response by disrupting the MHC class I (MHC-I) pathway, a critical mechanism for presenting viral antigens and activating cytotoxic T-cells [130]. MHC-I molecules present viral peptides to CD8^+^ T-cells, which are crucial for identifying and inducing apoptosis in infected cells [131]. However, SARS-CoV-2 ORF8 protein downregulates MHC-I expression through Beclin 1–mediated autophagy pathway, impairing the host’s ability to present viral antigens to cytotoxic T-cells and allowing the virus to evade immune detection [132]. This downregulation facilitates sustained viral replication and exacerbates infection severity. Moreover, gene expression profiling and biochemical analyses reveal that MHC-I expression is further suppressed by SARS-CoV-2 ORF6 protein, which inhibits NLRC5 both transcriptionally and functionally [133]. This suppression operates through the STAT1-IRF1-NLRC5 axis, where NLRC5 serves as a key transcriptional regulator of MHC class I expression.

### Mapping the Landscape of Coronavirus Immune Evasion

**Fig. 6:**
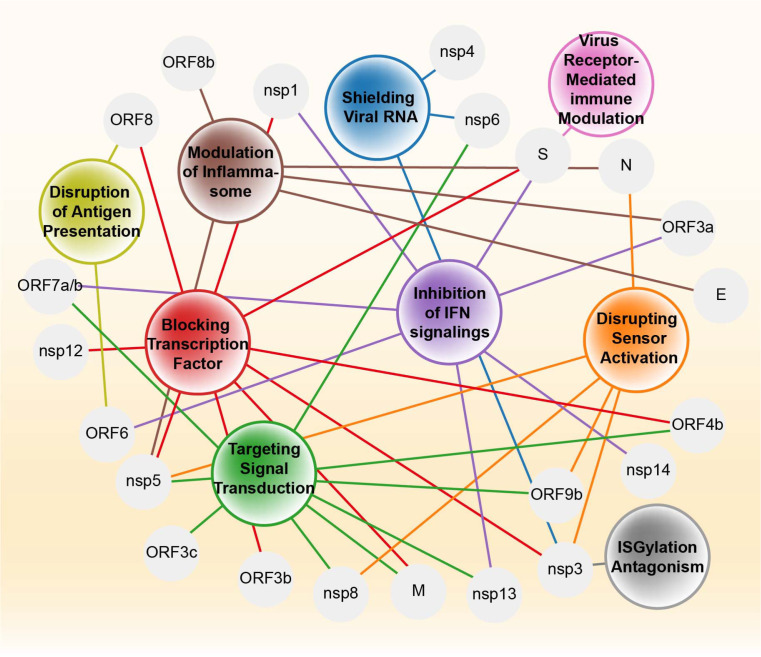
Multifaceted immune evasion strategies of highly pathogenic human coronaviruses. This schematic illustrates distinct mechanisms by which highly pathogenic human coronaviruses evade host innate immune responses. Each immune evasion strategy is represented by a colored circle, with viral proteins mapped to their respective roles through connecting lines. For a detailed breakdown of these strategies, see Table 1.

While maintaining a strategy-oriented framework organized around RLRs, TLRs, IFN signaling, and inflammasomes, we highlight overarching patterns of viral immune modulation, compare evolutionary strategies across these coronaviruses, and emphasize functional intersections of viral proteins that operate across multiple pathways. (Fig. 6). This integrative perspective allows us to connect individual molecular interactions to broader principles of immune evasion and pathogenesis. Recent reviews provide complementary analyses [134-136], and we build upon these works to identify gaps in current understanding and potential therapeutic implications.

To move beyond a descriptive summary of individual mechanisms, we sought to integrate these findings into a systems-level framework that captures the hierarchical and coordinated nature of coronavirus immune evasion. This approach enables the identification of key regulatory nodes and functional intersections that are repeatedly targeted across different viruses. This mapping highlights shared and virus-specific targeting patterns and provides a comparative framework for understanding the convergence of immune evasion strategies.

**Coordinated Immune Evasion Strategies:**

Integrating findings from SARS-CoV, MERS-CoV, and SARS-CoV-2 reveals that, although individual studies often emphasize single proteins or pathways, overlapping viral targets form a hierarchical network of immune evasion essential for viral fitness, with coronaviruses coordinating disruptions across sensor activation, signaling, transcriptional regulation, ISGylation, and antigen presentation (Figs. 2–5). Viral proteins such as ORF9b, nsp3, and ORF6 exemplify this interconnectedness by simultaneously blocking RIG-I, MAVS, TBK1, IRF3, and STATs, producing a cascade of immune paralysis, while central molecules like IRF3, MAVS, and STATs are repeatedly attacked by multiple viral proteins through diverse mechanisms, underscoring their vulnerability (Fig. 6 and Table 1). This redundancy likely reflects evolutionary pressure to ensure robust suppression of type I IFN responses. Beyond interferon antagonism, multifunctional proteins such as nsp3 and nsp5 span several immune evasion categories, accessory proteins like ORF8 specialize in disrupting antigen presentation, and inflammasome activators including ORF3a, E, and N proteins converge on the NLRP3 pathway to drive late-stage inflammation and immunopathology.

Notably, these observations suggest that coronavirus immune evasion is not random but follows a convergent strategy targeting a limited number of central signaling hubs, including MAVS, TBK1, IRF3, and STATs. Such convergence likely reflects evolutionary pressure to ensure robust suppression of antiviral responses. In this context, these host factors may represent critical vulnerability nodes and promising targets for therapeutic intervention. Importantly, these immune evasion strategies appear to be temporally coordinated. Early during infection, viral proteins predominantly suppress interferon production and signaling to facilitate viral replication. At later stages, however, certain viral components promote inflammasome activation and excessive inflammatory responses, contributing to immunopathology. This temporal dichotomy highlights the dual role of innate immunity in both antiviral defense and disease progression.

**Functional Versatility of Viral Proteins in Viral Fitness and Immune Escape:**

Several SARS-CoV-2 proteins display dual functionality, contributing both to viral replication and to the modulation of host immune responses. Nsp3 and nsp5, the principal viral proteases, are indispensable for viral polyprotein processing, and their intrinsic proteolytic activity also underlies potent immunomodulatory effects, cleaving host signaling proteins such as RIG-I, MAVS, and IRF3. Indeed, nsp3 and nsp5 cover the most diverse immune evasion strategies, highlighting their dual roles in both viral protein maturation and host immune suppression (Fig. 6). Their activities further extend to de-ISGylation, ISG15 deconjugation, and deubiquitination, thereby suppressing antiviral defenses.

In contrast, the replicase complex, composed of nsp12, nsp13, nsp14, nsp15, and nsp16, exhibits immunomodulatory effects that are distinct from its canonical functions, which primarily support viral RNA synthesis and modification. Although some of these proteins (*e.g.*, nsp12, nsp13, nsp14) have been implicated in immune interference, such as IFNAR1 downregulation or host translation shutdown [137], these effects appear secondary to their replication-related roles.

On the other hand, nsp1 and accessory proteins including ORF6, ORF8, and ORF9b are more directly engaged in immune suppression, targeting key host pathways such as interferon signaling, antigen presentation, and inflammasome activation, often independent of replication functions. Structural proteins (S, M, N, and E) also contribute to immune modulation, challenging the traditional view that immune evasion is confined to nonstructural proteins. As highlighted by Oliveira Silva Pinto *et al*. (2024), structural components act not merely as scaffolds but as active modulators of host antiviral defenses. Juxtaposing structural and nonstructural protein activities thus provides an integrated perspective on coronavirus strategies to suppress interferon responses and inflammasome activation.

**SARS-CoV-2 Variants and Immune Evasion:**

SARS-CoV-2 has undergone extensive genetic diversification, giving rise to multiple variants of concern (VOCs) such as Alpha (B.1.1.7), Delta (B.1.617.2), and Omicron (B.1.1.529) lineages, each exhibiting distinct mutational profiles that influence virus-host interactions [138]. These variants display differential capabilities to antagonize innate immune pathways, including type I IFN responses and inflammasome activation. For example, mutations in the S protein, such as D614G and P681R, have been linked to enhanced viral entry and altered sensitivity to IFN-mediated restriction [139].[Table T2]

Some groups have reported that mutations in viral proteins alter immune responses by disrupting the immune mechanisms normally maintained by the wild-type protein. SARS-CoV-2 variants carrying an nsp6 mutation characterized by a three–amino acid deletion within the 81–120 aa region, critical for autophagy induction and STING1 degradation, exhibit reduced autophagy, diminished STING1 degradation, and consequently enhanced host antiviral immunity [140]. In parallel, another study reported that the high frequency of nsp6 mutations alters its structural integrity, thereby impairing type I IFN signaling [141]. A truncated ORF7b variant (Δ382 strain identified in Singapore & Taiwan in 2020), associated with milder disease, has lost its ability to suppress IFN production [51]. The ORF6 D61L mutation, which emerged in Omicron BA.2 and BA.4, reduces inhibition of STAT1/STAT2 nuclear import compared with the ancestral strain, thereby weakening immune evasion [80]. Variant-dependent differences have also been observed in the ability of the S protein to downregulate hACE2, which correlates with disease severity; notably, Omicron BA.1 shows markedly reduced hACE2 downregulation compared with the ancestral, Alpha, Beta, Gamma, and Delta variants, consistent with its milder clinical presentation [116]. This effect may also be linked to dysregulation of the renin–angiotensin system, thereby influencing inflammation.

While many mutations diminish immune antagonism, ORF3b variants identified in Ecuador with extended length due to loss of a premature stop codon (e.g., GISAID: EPI_ISL_422564 and EPI_ISL_422565) exhibit significantly enhanced anti-IFN-I activity relative to the ancestral strain [71]. Collectively, these findings suggest that SARS-CoV-2 evolution has refined viral strategies to modulate host immunity through both conserved and variant-specific mechanisms. Integrating variant-associated immune modulation into our understanding of coronavirus pathogenesis provides important insight into differential disease outcomes and may guide the development of variant-resilient antiviral strategies. Notably, the current understanding of MERS-CoV immune evasion remains relatively limited compared to that of SARS-CoV and SARS-CoV-2. While several key mechanisms, such as interference with TBK1/IKKε signaling and suppression of interferon responses, have been identified, many aspects of MERS-CoV-mediated modulation of inflammasomes, ISGylation, and antigen presentation remain poorly defined. Future studies addressing these gaps will be essential for achieving a more comprehensive and balanced understanding of coronavirus immune evasion.

## Summary/Conclusion

Highly pathogenic human coronaviruses employ a broad spectrum of immune evasion strategies that target RLRs, TLRs, IFN signaling, inflammasomes, ISGylation, and antigen presentation pathways. These mechanisms not only facilitate viral replication but also contribute to immune-mediated pathology. Importantly, their relative contributions to disease pathogenesis are not equivalent.

Among these strategies, suppression of type I IFN signaling, via disruption of IRF3, STAT1/2, and IFNAR1, is established as a principal driver, closely associated with enhanced viral replication and increased disease severity. Several viral proteins, including ORF9b and nsp3, act on multiple nodes within the RLR and TLR pathways, underscoring their central role in dampening host antiviral defenses. Comparative analyses of SARS-CoV, MERS-CoV, and SARS-CoV-2 reveal that, although the precise molecular interactions differ, a conserved strategy of convergent inhibition targeting key adaptors such as MAVS and TBK1 is maintained (Fig. 2). Within IFN signaling, numerous non-structural and accessory proteins exert overlapping inhibitory effects, including suppression of Interferon-stimulated gene factor 3 (ISGF3) complex activation and blockade of their nuclear translocation [66, 75-77, 79, 80, 83] (Fig. 3). Collectively, these findings identify viral proteins such as nsp1 and ORF6 as central hubs mediating broad immunosuppressive effects across multiple antiviral pathways.

Inflammasome modulation is frequently coordinated with inhibition of RLR and IFN pathways, reflecting a broader strategy of simultaneous interference with multiple innate immune arms. Although the specific inflammasome components targeted differ among SARS-CoV, MERS-CoV, and SARS-CoV-2, the functional outcome, dysregulation of pro-inflammatory and antiviral responses, remains consistently observed. Importantly, many of these effects are highly context-dependent, with viral proteins exerting distinct influences depending on cellular environment and stress conditions. For instance, while the SARS-CoV-2 N protein directly enhances NLRP3–ASC interactions to promote inflammasome assembly [89], other proteins act in a more conditional manner: the E protein modulates activation through ion channel activity and ER stress responses [100, 101], whereas SARS-CoV ORF8b activates NLRP3 via lysosomal stress and autophagy dysregulation [90]. This underscores that coronavirus inflammasome regulation is not uniform but instead shaped by context-specific mechanisms that fine-tune host immune outcomes.

Additional immunomodulatory mechanisms include receptor-mediated effects (hACE2, hDPP4, DC-SIGN, L-SIGN), disruption of the renin–angiotensin system by SARS-CoV and SARS-CoV-2 [109, 115], and deconjugation of ISG15 by viral PLpro enzymes [126, 127], which undermines antiviral ISGylation. SARS-CoV-2 further impairs MHC-I antigen presentation via ORF8 and ORF6, reducing CD8^+^ T-cell recognition and promoting viral persistence [132, 133]. These mechanisms appear to function as ancillary strategies that complement primary immune evasion pathways.

Taken together, these observations underscore the importance of viewing coronavirus immune evasion as a multilayered network in which viral proteins act redundantly or synergistically. This perspective highlights critical viral determinants that warrant prioritization for therapeutic targeting and mechanistic investigation.

For instance, nsp3 and nsp5 are essential not only for processing viral polyproteins but also for antagonizing virus-induced innate immunity, making them compelling druggable targets. A recent study demonstrated that GRL-0617, identified through screening of approved drug libraries, inhibited SARS-CoV-2 PLpro activity as well as its ISGylation function [142]. Structural analysis revealed that GRL-0617 binds to the ubiquitin-specific protease (USP) domain of PLpro.

By contrast, interferon-based therapies including IFN-2b, IFN-α, PEG-IFN-α1a and inflammatory cytokine modulators such as anti-IL-1 and anti-IL-6 agents have shown limited or contradictory efficacy [143]. The failure of these conventional immune-suppressive strategies further underscores the need to prioritize direct targeting of viral determinants and to advance mechanistic investigations into their roles in immune evasion.

Despite extensive research, key questions remain regarding the trade-offs between immune evasion and host damage, the impact of multitargeting on viral fitness versus inflammation, the *in vivo* hierarchy of evasion mechanisms, and the balance between immune modulation and replication. Addressing these issues will require variant-specific studies, temporal mapping of viral protein–host interactions, and the identification of conserved evasion nodes, with multi-omics and structural biology approaches essential for deeper mechanistic insight. Importantly, the uneven depth of knowledge among SARS-CoV, MERS-CoV, and SARS-CoV-2 highlights a critical need for expanded research on less-studied coronaviruses, particularly MERS-CoV. Addressing these gaps will not only refine our comparative understanding but also improve preparedness for future emerging coronaviruses.

## Figures and Tables

**Table 1:** Mechanistic roles of viral proteins in modulating host immune responses.

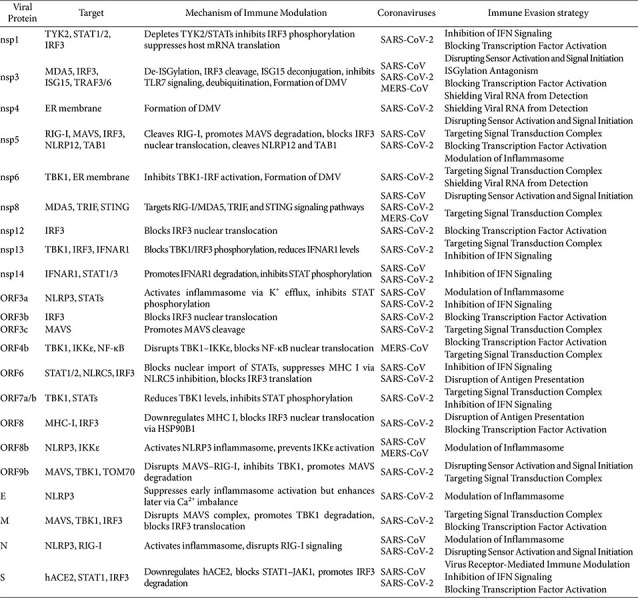

**Table 2 T2:** Variant-specific mutations affecting immune evasion.